# The spectraplakin Short stop is an essential microtubule regulator involved in epithelial closure in *Drosophila*

**DOI:** 10.1242/jcs.193003

**Published:** 2017-02-15

**Authors:** Zsanett Takács, Ferenc Jankovics, Péter Vilmos, Péter Lénárt, Katja Röper, Miklós Erdélyi

**Affiliations:** 1 Institute of Genetics, Biological Research Centre of the Hungarian Academy of Sciences, Temesvári krt. 62, Szeged 6726, Hungary; 2 Cell Biology and Biophysics Unit, European Molecular Biology Laboratory (EMBL), Meyerhofstrasse 1, Heidelberg 69117, Germany; 3 MRC-Laboratory of Molecular Biology, Cambridge Biomedical Campus, Francis Crick Avenue, Cambridge CB2 0QH, UK

**Keywords:** *Drosophila*, Spectraplakin, Short stop, Dorsal closure, Microtubule, Actin

## Abstract

Dorsal closure of the *Drosophila* embryonic epithelium provides an excellent model system for the *in vivo* analysis of molecular mechanisms regulating cytoskeletal rearrangements. In this study, we investigated the function of the *Drosophila* spectraplakin Short stop (Shot), a conserved cytoskeletal structural protein, during closure of the dorsal embryonic epithelium. We show that Shot is essential for the efficient final zippering of the opposing epithelial margins. By using isoform-specific mutant alleles and genetic rescue experiments with truncated Shot variants, we demonstrate that Shot functions as an actin–microtubule cross-linker in mediating zippering. At the leading edge of epithelial cells, Shot regulates protrusion dynamics by promoting filopodia formation. Fluorescence recovery after photobleaching (FRAP) analysis and *in vivo* imaging of microtubule growth revealed that Shot stabilizes dynamic microtubules. The actin- and microtubule-binding activities of Shot are simultaneously required in the same molecule, indicating that Shot is engaged as a physical crosslinker in this process. We propose that Shot-mediated interactions between microtubules and actin filaments facilitate filopodia formation, which promotes zippering by initiating contact between opposing epithelial cells.

## Abstract (precis)

**Summary:** The *Drosophila* spectraplakin Shot coordinates actin–microtubule interactions at the leading edge to ensure the formation of filopodia that promote the dorsal closure of the embryonic epithelium.

## INTRODUCTION

Towards the end of *Drosophila* embryogenesis, an epithelial discontinuity is formed at the dorsal surface of the embryo, which is covered by amnioserosa cells. During dorsal closure, this gap is closed by the dorsal epithelium while the amnioserosa disintegrates. Dorsal closure involves two distinct mechanisms: convergence of two opposed epithelial sheets towards the dorsal side, and subsequent zippering of the hole at the dorsal midline of the embryo (Jacinto et al., 2002). At the onset of the closure, cells in the first row of the embryonic epithelium differentiate into dorsal-most epithelial (DME) cells and establish a straight movement front, which initiates dorsal-ward migration. Migrating epithelial sheets first meet at the anterior- and posterior-most ends of the dorsal opening where they fuse by a zipper-like mechanism. Fusion of the sheets spreads from the two zippering corners towards the center of the opening, resulting in a typical lens-shaped outline of the dorsal hole throughout the entire process. During zippering, interacting surfaces of opposing DME cells form lamellar overlaps, which are resolved by shortening and concomitant thickening to achieve seamless closure of the dorsal epithelium (Eltsov et al., 2015).

Genetic and biophysical investigations revealed that the dorsal opening has to be closed in a tightly regulated and efficient manner (Hutson et al., 2003). Several forces provided by various tissues contribute to the closure process, and loss of one of these forces can be compensated for by the others. In these cases, the dorsal opening is sealed, but the dynamics of the closure is abnormal. Mutations leading to abnormal closure dynamics – although not necessarily causing morphological abnormalities – might have evolutionarily relevance.

Efficient dorsal closure requires the dynamic rearrangement of the cytoskeleton in epithelial cells (Martin and Parkhurst, 2004). DME cells form a leading edge facing towards the dorsal opening, where they accumulate an actomyosin cable. In addition, DME cells extend actin-rich cellular protrusions, such as filopodia and lamellipodia, mediating the initial contact between the opposing DME cells. Dynamic filopodia are essential both for the mechanics of epithelial adhesion during dorsal closure and for the correct ‘matching’ of opposing cells (Hakeda-Suzuki et al., 2002; Harden et al., 1999; Jacinto et al., 2000; Jankovics and Brunner, 2006; Woolner et al., 2005).

The microtubule (MT) network has also been demonstrated to rearrange during dorsal closure (Jankovics and Brunner, 2006; Kaltschmidt et al., 2002). At the onset of closure, DME cells display an irregularly distributed network of MTs. During closure, MTs reorganize to form acentrosomal bundles that are aligned along the dorsal–ventral cell axis. Although the bundles are stable, individual MTs remain highly dynamic, and at the leading edge they grow into cell protrusions (Jankovics and Brunner, 2006). MTs are dispensable for dorsal-ward migration of the epithelia but contribute to efficient zippering of the epithelial hole by two distinct mechanisms at two consecutive steps. In the early zippering phase, MTs at the leading edge regulate protrusion dynamics to promote initial interactions between DME cells. During later stages of zippering, shrinking MTs are attached with their plus-ends to newly formed cell adhesions, where they are thought to provide a MT motor-based force to resolve areas of the opposing DME cells that have overlapping lamellae (Eltsov et al., 2015).

During morphogenesis, not only the proper organization of the MT and actin networks but also the coordination of their interactions is essential for dynamic cell shape changes. In the past years, an expanding number of proteins and protein complexes mediating structural connections between actin and MTs have been described, among which spectraplakins represent an important class of evolutionarily conserved actin–MT crosslinkers (Coles and Bradke, 2015; Suozzi et al., 2012).

Spectraplakins can interact with MTs through two distinct domains localized at their C-terminus. The C-tail and the GAS2 domains mediate the association of spectraplakins with growing plus-ends and along the MT lattice, respectively (Alves-Silva et al., 2012; Applewhite et al., 2010). The C-tail domain contains three MT tip localization signals (MtLS), which target spectraplakin to the MT plus-end by linking it to EB1 (also known as MAPRE1 in mammals), a core component of the plus-end tip complex. At the N-terminal end of spectraplakins, two calponin homology (CH) domains facilitate binding to the actin network. The actin- and MT-interacting domains are separated by a flexible rod domain consisting of a series of spectrin repeats. This structural arrangement of the binding domains enables crosslinking of actin with the growing MT tip or with the MT lattice. Adjacent to the GAS2 domain, spectraplakins possess EF-hand motifs, which regulate autoinhibition of the protein established through an interaction between its N-terminal CH and C-terminal GAS2 domains (Applewhite et al., 2013). In addition to their actin- and MT-interacting domains, spectraplakins harbor a plakin domain enabling their direct binding to adhesive junctions (Röper and Brown, 2003). Through alternative splicing and alternative promoter usage, an enormous number of different protein isoforms, with a wide variety of domain compositions, can be created from a single spectraplakin gene, which enables the spectraplakins to fulfill a wide array of functions in various biological processes (Hahn et al., 2016; Röper and Brown, 2003; Röper et al., 2002).

While vertebrates have two spectraplakins, ACF7 (also known as MACF1) and BPAG1 (also known as DST), the *Drosophila* genome contains a single spectraplakin homolog *short stop* (*shot*) providing a powerful model for studying spectraplakin function (Hahn et al., 2016). In *Drosophila*, Shot has been shown to be involved in a wide variety of developmental processes, such as neuronal cell growth, tracheal cell fusion and attachment of tendon cells to the muscles. In each case, Shot mediates cytoskeletal rearrangements; however, the exact requirements of its individual functional activities in a given cell type are defined by the cellular context (Bottenberg et al., 2009; Lee and Kolodziej, 2002a; Sanchez-Soriano et al., 2009; Subramanian et al., 2003).

Despite the substantial progress achieved in understanding the functions of spectraplakins, the way these activities are regulated is still not completely clear. The analysis of spectraplakins is complicated by the variability of domain composition, the complex interactions between the domains and the enormous differences in the cytoskeletal organization of the cell types in which spectraplakin function has been studied. Therefore, even functions of individual domains seem to vary between experimental conditions, which means conclusions in one system cannot be applied to other model systems. In addition, analysis of cytoskeletal processes in the intact animal in an unperturbed developmental context is not always possible at an appropriate spatial and temporal resolution. Dorsal closure of the embryonic epithelium in *Drosophila*, however, provides an excellent model to investigate spectraplakin function. In this study, we demonstrate that Shot is required for the final sealing of the epithelial sheets during dorsal closure. In DME cells, Shot acts as a MT–actin crosslinker to stabilize MTs and regulate proper formation of the MT network. At the leading edge, Shot-mediated MT–actin interactions promote formation of filopodia required for the initial contact between opposing DME cells during zippering.

## RESULTS

### Shot is required for efficient zippering of the epithelial sheets during dorsal closure

**Fig. 1.**
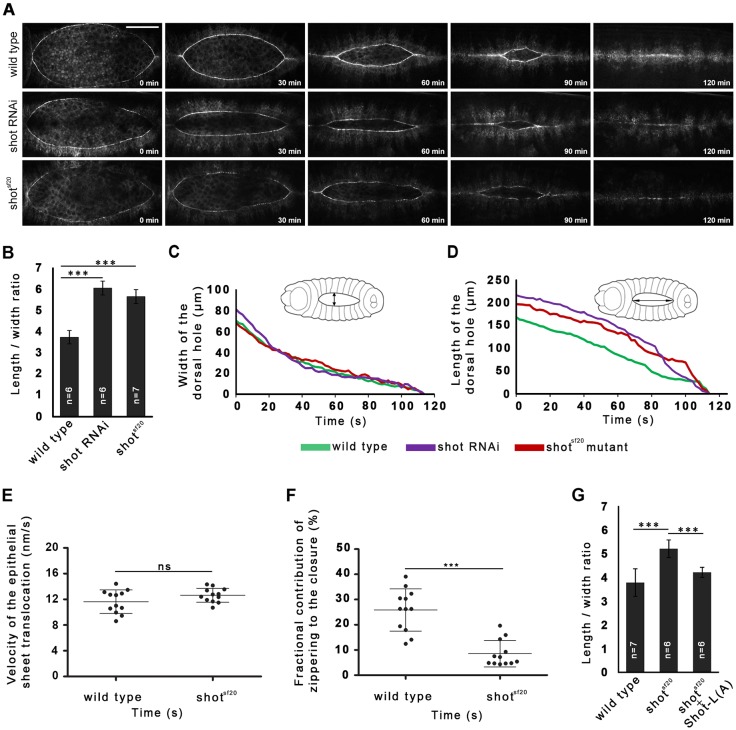
**Shot is required for efficient zippering of the dorsal hole in *Drosophila* embryos.** (A) Dorsal closure phenotype in wild-type, *shot* RNAi and *shot^sf20^* mutant embryos. The first cell row of the epithelial sheets is highlighted by Zasp52^ZCL0432^–EGFP. Scale bar: 50 µm. (B) Quantification of the zippering efficiency of the dorsal opening in wild-type, *shot* dsRNA-treated, and *shot^sf20^* mutant embryos. The length-to-width ratio of the dorsal opening when it is at a 30 µm width is shown. (C,D) Graphs showing closure kinetics of the dorsal hole in a wild-type (green), a *shot* dsRNA-injected (purple) and a *shot^sf20^* mutant embryo (red). For each category, data from an individual representative embryo are shown. (C) Width of the dorsal hole over time for the indicated embryos, representing the maximal distance between the converging epithelial sheets (double arrow). (D) Length of the dorsal hole over time for the indicated embryos, representing the maximal distance between zippering ends (double arrow). (G) Targeted expression of Shot-L(A)–GFP in the dorsal epithelium rescued the zippering defects of the *shot^sf20^* null mutant embryos. Mean±s.d. is shown for all quantitative data. ***P<0.001; ns, not significant (*t*-test).

To uncover new genes involved in cytoskeletal reorganization and function during dorsal closure, various cytoskeletal regulator genes were silenced by microinjection of embryos with *in vitro* synthesized double-stranded (ds)RNAs (Table S1). The EGFP signal of the ZASP52^ZCL423^ protein trap line was used to highlight the outline of the dorsal opening. *In vivo* imaging of the closure process revealed that, of the genes tested, only the silencing of *shot* results in abnormal dorsal closure (Fig. 1A; Movie 1). In embryos with reduced *shot* function, the opening is closed completely, but the dynamics of the closure is abnormal. In the *shot* RNA interference (RNAi) embryos, the dorsal opening was abnormally narrow, and not the typical wild-type teardrop-shaped dorsal hole. To confirm the RNAi results, the *shot^sf20^* mutant allele was used, which behaves genetically as a null allele and has been suggested to abolish all *shot* functions (Fig. S1). *shot^sf20^* homozygous embryos displayed the same abnormal closure phenotype as that induced by silencing *shot* (Fig. 1A; Fig. S2). To quantify the abnormal shape of the dorsal hole, the length-to-width ratio of the opening was used as a numeric parameter. The phenotype was most obvious towards the middle of the closure; therefore, the closure stage at 30 μm opening width was arbitrarily chosen to quantitatively characterize closure defects. In embryos injected with *shot* dsRNA and in *shot^sf20^* null mutants, the length-to-width ratio significantly increased indicating that *shot* plays an essential role during the closure of the dorsal hole (Fig. 1B).

The abnormally narrow shape of the opening can arise when the opposed epithelial sheets converge normally while the final zippering of the sheets is affected. To characterize the abnormal dynamics phenotype, a mathematical model of dorsal closure was applied (Hutson et al., 2003). In *shot* mutants, the length and width of the dorsal opening were measured throughout the entire closure process (Fig. 1C,D). The velocity of the epithelial sheet translocation (*v*), as well as the fractional contribution of zippering (*f*_z_) to the velocity of the closure was calculated. The null mutation of *shot* resulted in a decrease of *f*_z_ demonstrating that *shot* function is essential for efficient zippering (Fig. 1E,F).

To rescue the *shot* mutant phenotype, a GFP-tagged long isoform of Shot [Shot-L(A)–GFP] was used, which is the longest available transgenic Shot protein version and has been reported to rescue *shot* mutant phenotypes during embryogenesis (Bottenberg et al., 2009; Lee and Kolodziej, 2002b). The Shot-L(A)–GFP protein was expressed throughout the dorsally migrating epithelium of *shot* mutant embryos by the *pnr*-Gal4 driver, and the outline of the dorsal opening was highlighted by mCherry–Moesin. Targeted expression of the long Shot-L(A)–GFP protein rescued the zippering defect of the *shot* mutants, as demonstrated by the restoration of the length-to-width ratio of the dorsal opening to the wild-type level (Fig. 1G).

### Shot acts as an actin–MT cross linker in mediating zippering

**Fig. 2.**
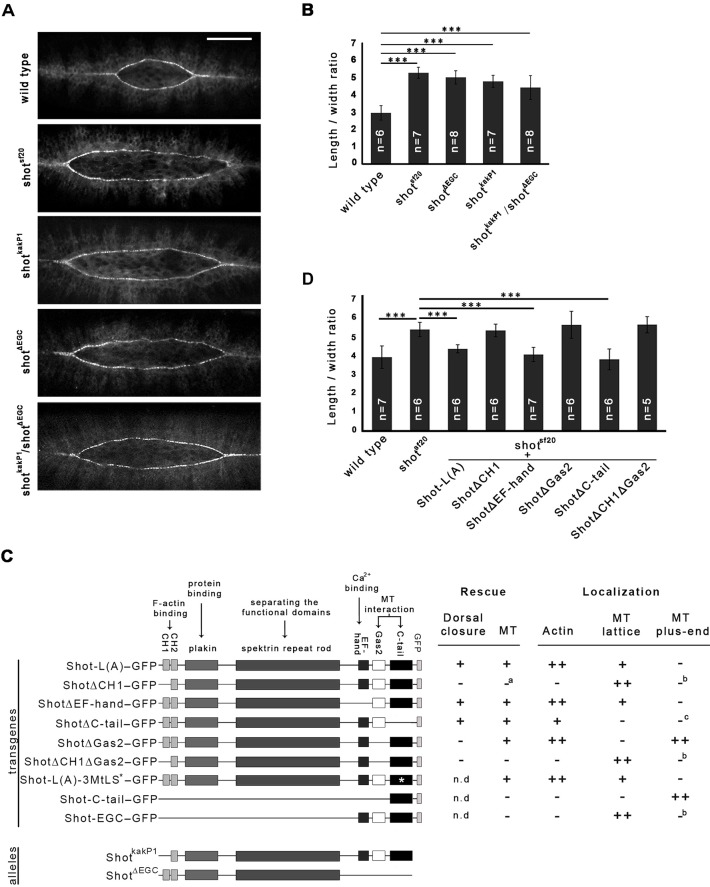
**MT-binding and actin-binding activities of Shot are essential for efficient zippering of the dorsal hole in *Drosophila* embryos.** (A) Zippering deffects of *shot^sf20^*, *shot^kakP1^*, *shot^ΔEGC^* and *shot^ΔEGC^/shot^kakP1^* mutant embryos at a closure stage of 30 µm hole width. Scale bar: 50 µm. (B) Length-to-width ratio of the dorsal opening when it is at a 30 µm width in various *shot* mutants. (C) Schematic representation of the longest available transgenic Shot protein version [Shot-L(A)–GFP] and its deletion constructs. *, mutated MtLS motifs. Rescue section: –, no rescue; +, rescue; *a*, for the lack of rescue, see explanation in the text. Localization section: –, no localization; +, weak localization; ++ strong localization; *b*, strong localization to the MT lattice may mask plus-tip localization; *c*, diffuse cytoplasmic localization may mask faint localization to the MTs. (D) Length-to-width ratio of the dorsal hole when it is at a 30 µm width in transgenic rescue experiments. Truncated versions of Shot-L(A)–GFP lacking various protein domains were expressed in the epithelia of *shot^sf20^* null mutant embryos. Mean±s.d. is shown for all quantitative data. ***P<0.001 (*t*-test).

In order to understand the role of the individual protein domains of Shot in dorsal closure, we investigated the mutant phenotypes of various *shot* mutant alleles abolishing distinct Shot activities. In addition, we carried out a detailed structure–function analysis of Shot using a series of *shot* transgenes in rescue experiments. Hence, GFP-tagged truncated versions of the Shot protein were expressed in the epithelia of *shot^sf20^* null mutant embryos by the *pnr*-Gal4 driver (Fig. 2C).

To investigate the requirement for the actin-binding activity of Shot, we made use of *shot^kakP1^* mutant embryos, which express Shot isoforms lacking the actin-binding CH1 domain (Fig. S1) (Gregory and Brown, 1998; Lee et al., 2000; Röper et al., 2002). Loss of the CH1 domain alone has previously been shown to lead to loss of F-actin-binding ability from the N-terminus of Shot (Lee and Kolodziej, 2002b). In *shot^kakP1^* mutants, zippering of the dorsal hole was delayed, indicating the requirement for the actin-binding activity of Shot in zippering (Fig. 2A,B; Movie 1). To further investigate the contribution of the actin-organizing activity of Shot to closure, we used a transgene lacking the CH1 domain, mimicking the *shot^kakP1^* mutant allele. Confirming our results obtained with *shot^kakP1^*, the shotΔCH1–GFP transgene failed to rescue the *shot* mutant zippering defect (Fig. 2D). Therefore, we conclude that association of Shot with actin filaments is essential for efficient zippering of the opposing epithelial sheets.

To gain a deeper insight into Shot function during dorsal closure, we studied the requirement for its MT-organizing activity. Crispr/Cas9-based genome editing was applied to generate a novel *shot* mutant allele specifically impairing its binding to MTs (Table S2). We generated an 1895 bp genomic deletion, which removes the coding sequence of the EF-hand and the Gas2 domains, and, after four additional amino acids, it ends in a premature stop codon. The resulting truncated protein, which we designated Shot^ΔEGC^, lacks the EF-hand, the Gas2 and the C-tail domains, leading to complete loss of the MT-binding activity. Expression of the truncated protein in DME cells was confirmed by immunostaining of *shot^ΔEGC^* mutant embryos using a polyclonal antibody raised against the spectrin repeats (Fig. S1). In *shot^ΔEGC^* mutant embryos, we detected the same zippering defect as in the *shot^sf20^* null mutants and the *shot^kakP1^* actin-binding-deficient mutants, suggesting that both the MT-binding and actin-binding activities of Shot are essential for efficient zippering of the epithelial sheets (Fig. 2A,B).

The analysis of *shot^ΔEGC^* mutant embryos revealed that the C-terminal region composed of the EF-hand, Gas2 and C-tail domains is required for zippering. To further dissect the requirement for the C-terminal region of Shot, we carried out rescue experiments in the *shot^sf20^* null mutant background with transgenes lacking one of these domains (Fig. 2C). Targeted expression of the Shot protein lacking the EF-hand domain (ShotΔEF-hand–GFP) restored the zippering efficiency of *shot^sf20^* mutants to the wild-type level, indicating that this domain is not essential for zippering (Fig. 2D). Next, we expressed a truncated Shot protein version lacking the C-tail domain (ShotΔC-tail–GFP), which has been shown to mediate the association of Shot with the growing MT plus-ends by interacting with EB1. In this case, we detected a rescue of the *shot* mutant zippering phenotype, indicating that interaction of Shot with EB1 and its accumulation at the MT plus-tips is dispensable for dorsal closure (Fig. 2D). The Shot protein variant lacking the MT-stabilizing Gas2 domain (ShotΔGas2–GFP), however, failed to rescue the zippering defect (Fig. 2D). These findings are consistent with the data obtained with the isoform-specific mutant allele *shot^ΔECG^*, and indicate that it is not the EF-hand or the C-tail domain but rather Gas2 that is required in this cellular context. Expression of a transgenic protein lacking both the CH1 and the Gas2 domains (ShotΔCH1ΔGas2–GFP) failed to rescue dorsal closure defects of the null mutant, confirming the requirement for the actin- and MT-binding activities of Shot (Fig. 2D).

In *shot^ΔECG^*/*shot^kakP1^* embryos, Shot variants contain exclusively either CH1 or Gas2, but none of them has actin- and MT-binding activities simultaneously. These embryos exhibited the zippering defect, indicating that both the actin- and the MT-binding domains have to be present in the same Shot molecule for proper dorsal closure (Fig. 2A,B; Movie 1).

Taken together, these results demonstrate that both the actin- and MT-binding activities of Shot are required for dorsal closure, and suggest that Shot acts as an actin–MT cross-linker to mediate the zippering step of dorsal closure.

### Shot regulates MT organization in epithelial cells during dorsal closure

**Fig. 3.**
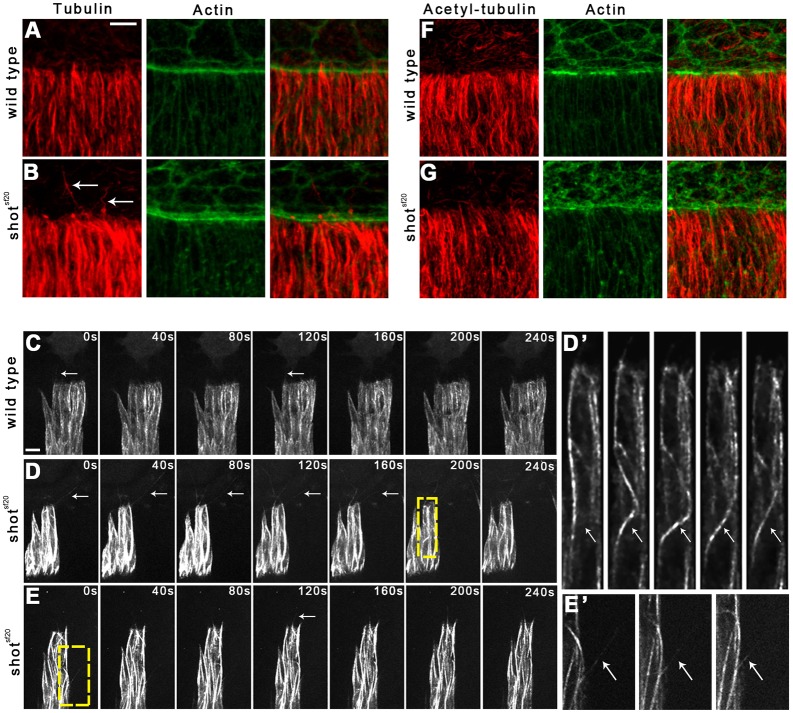
**Abnormal MTs in the *shot^sf20^*mutant DME cells.** (A,B) Immunofluorescence staining of wild-type (A) and *shot^sf20^* mutant (B) embryos at the dorsal closure stage. Tubulin staining labels the MT network (red); actin is labeled with phalloidin (green). White arrows indicate abnormally long and curled MTs at the leading edge of *shot^sf20^*mutant DME cells. (C,D) Frames taken from movies showing Tubulin–EGFP-expressing DME cells in wild-type (C) and in *shot^sf20^*mutant (D,E) embryos. White arrows indicate MTs growing into protrusions. MTs are abnormally long and curled at the leading edge of *shot^sf20^*mutant DME cells. (D′,E′) Enlargement of the boxed regions in D and E showing bending of a MT in D′ and protrusion of a MT at the lateral surface in E′. (F,G) Staining for acetylated tubulin labels stabilized MTs (red); actin is labeled with phalloidin (green). In *shot^sf20^* mutants, no abnormal distribution of stabilized MTs is detectable. Scale bars: 5 µm (C,D,E); 10 µm (A,B,F,G).

The MT network has been shown to reorganize during dorsal closure, and its function is required for the zippering of the epithelial sheets (Eltsov et al., 2015; Jankovics and Brunner, 2006; Kaltschmidt et al., 2002). To study the involvement of *shot* in MT network organization, we examined MT distribution in *shot* mutant epithelial cells via immunohistochemical labeling. In the cell body of *shot^sf20^* null mutants, the overall morphology of the MT network appeared to be slightly disorganized (Fig. 3A,B). At the leading edge of mutant DME cells, MTs intruded into filopodia-like protrusions; however, the MTs were frequently abnormally long and bent, indicating that *shot* regulates their proper organization in the cell body and at the leading edge.

To gain a more detailed insight into the MT organization of the *shot* mutant DME cells, live imaging of embryos expressing Tubulin–EGFP was performed. In the cell body of *shot^sf20^* mutants, sudden bending of MTs was detected (Fig. 3D,D′). Moreover, MTs frequently protruded at the lateral surface of the epithelial cells (Fig. 3E,E′; Movie 2). At the leading edge of the mutant DME cells, long and bent MTs protruded from the cell body over the amnioserosa cells (Fig. 3D). The emergence of abnormal MTs coincided with the onset of the zippering stage, and they extended throughout the entire leading front of the dorsal epithelium (Movie 3). Time-lapse analysis revealed that the maximum length of the protruding MTs was higher in the *shot^sf20^* mutants than in wild type (3.8±1.1 μm in wild type, *n*=108, versus 6.8±3.5 μm in *shot^sf20^* mutants, *n*=109; mean±s.d.; *P*<0.001, *t*-test), indicating that Shot plays a role in regulating MT stability.

In mutant cells, altered MT dynamics, such as faster polymerization rate or lower catastrophe frequency, can lead to longer MTs which, having reached the cell cortex, continue to grow and push out the cell cortex or bend backward. To study MT stability in epithelial cells, we investigated the stabilization state of the MTs by immunolabeling acetylated tubulin. Acetylation of α-tubulin is a post-translational modification found mainly on long-lived MTs and serves as a hallmark of stable MTs. Consistent with previous findings, we detected acetylated tubulin incorporated into parallel MT bundles in the cell body of wild-type DME cells (Fig. 3F) (Wolf et al., 1988). Using immunohistochemical labeling of acetylated tubulin, we were unable to detect any abnormal distribution of acetylated MTs in *shot^sf20^* mutant DME cells (Fig. 3G). Abnormal MTs present at the leading edge of *shot* mutants were not labeled by anti-acetylated tubulin antibody indicating that Shot regulates the dynamic pool of MTs.

**Fig. 4.**
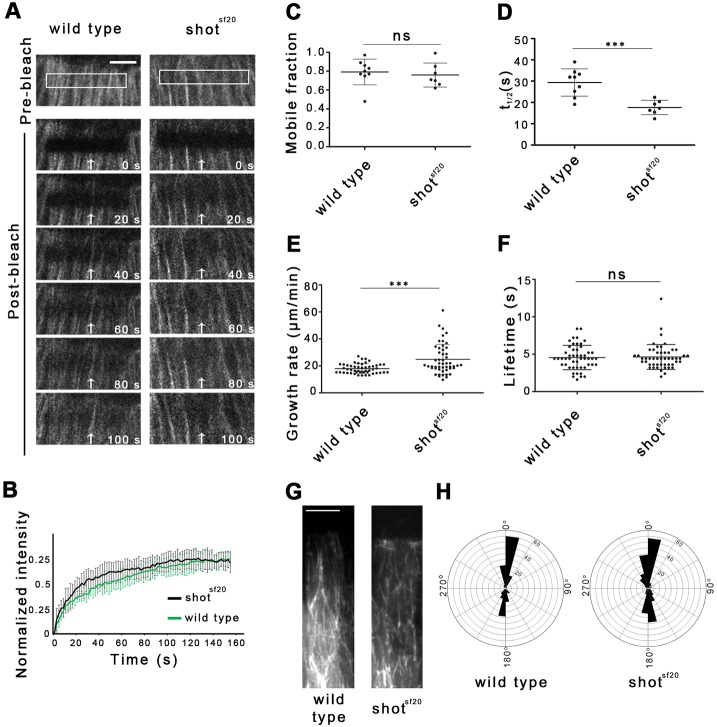
**Shot stabilizes dynamic MTs of DME cells by regulating their dynamic properties.** (A) Movies showing recovery of Tubulin–EGFP fluorescence in wild-type and *shot^sf20^* mutant DME cells in a representative FRAP experiment. White boxes and white arrows indicate photobleached regions. Scale bar: 5 µm. (B) FRAP recovery curves show the relative GFP fluorescence intensities within the photobleached regions in wild-type (black, *n*=9) and *shot^sf20^* mutant (gray, *n*=7) DME cells. (C,D) Scatter dot plots of fluorescence recovery halftimes (*t*_1/2_) and mobile fractions of Tubulin–EGFP measured in wild-type and *shot^sf20^* mutants. In *shot^sf20^* mutants, *t*_1/2_ was increased, whereas the mobile fraction of Tubulin–EGFP was unaffected. (E,F) Scatter dot plots of MT growth rate and lifetime of EB1 comets in wild-type (*n*=56 comets in two cells from two embryos) and *shot^sf20^* mutant (55 comets in two cells from two embryos) DME cells expressing EB1–EGFP. Displacement of EB1–EGFP comets was faster in *shot^sf20^* mutants than in wild-type DME cells, whereas the life-time of comets was unaffected. In C–F, results are mean±s.d. ***P<0.001; ns, not significant (*t*-test). (G) Projections of ten consecutive time frames of a movie showing EB1–EGFP tracks in wild-type and *shot^sf20^* mutant DME cells. The projected time-lapse spans 11 s. (H) Windrose plots of MT growth tracks in wild-type (*n*=181 in four cells from two embryos) and *shot^sf20^* mutant DME cells (*n*=269 in four cells from two embryos) expressing EB1–EGFP.

Fluorescence recovery after photobleaching (FRAP) assays were applied to analyze the turnover of tubulin, which reflects the dynamic properties of the MTs. In living embryos, EGFP-tagged β-tubulin was constitutively expressed, which incorporated into the MTs. A 2 μm-wide stripe close to the leading edge of the DME cells was bleached, and the recovery of Tubulin–EGFP fluorescence was imaged by *in vivo* video microscopy (Fig. 4A). The mobile fraction, reflecting the proportion of proteins undergoing turnover in the MTs, and the recovery half-time, reflecting turnover speed, were determined (Fig. 4B–D). A quantitative analysis of the FRAP curves revealed that the mobile fraction of tubulin was 0.8±0.06 (mean±s.d.) in the wild-type epithelial cells. In mutant DME cells, a similar mobile fraction was detected (0.76±0.13), confirming the finding that Shot does not influence the stabilized subset of the MTs, but rather exerts its function on the dynamic subset (Fig. 4C). Indeed, the recovery half-time of Tubulin–EGFP (*t*_1/2_) decreased to 17.6±3.4 s compared to the wild-type value of 29.3±6.4 s (mean±s. d.) indicating a faster turnover of Tubulin–EGFP between the MTs and the cytosol (Fig. 4D). Thus, MTs in *shot* mutants are more dynamic, suggesting that Shot affects the MT organization of DME cells by regulating the dynamic properties of MTs.

Faster turnover of Tubulin–EGFP in *shot* mutants could be a consequence of alterations in the parameters of plus-end dynamics, such as growth speed or time spent on growth. To test the function of Shot in MT growth regulation, EB1–EGFP was expressed in *shot^sf20^* null mutant epithelial cells (Movie 4). EB1 binds to polymerizing MT plus-ends, which enables direct measurement of the dynamic instability parameters by *in vivo* imaging. In mutant cells, the growth rate of MTs reflected by the speed of EB1 comets increased significantly. Instead of the wild-type growth rate of 17.9±3.5 μm/min (mean±s.d.), we measured 24.8±4.6 μm/min in the mutant cells, supporting the finding that Shot regulates MT dynamics (Fig. 4E). Quantitative analysis of EB1 tracks revealed that the lifetime of EB1 comets remained unaffected in *shot^sf20^* mutants indicating that the catastrophe frequency is not influenced by Shot activity (Fig. 4F).

It has been previously shown that spectraplakins can cross-link growing MT tips to actin filaments, which determine the growth direction of individual MTs (Kodama et al., 2003). To test this activity of Shot, we tracked EB1 comets in the cell body of epithelial cells. In both wild-type and *shot^sf20^* mutant embryos, most of the MTs polymerized parallel to the long axis of DME cells, indicating that Shot is not required for the regulation of MT growth direction along the actin filaments (Fig. 4G,H).

Taken together, these results demonstrate that Shot regulates the morphology of the microtubule network by stabilizing the dynamic microtubules in the DME cells.

### MT-binding activity of Shot is required but is not sufficient for MT stabilization

**Fig. 5.**
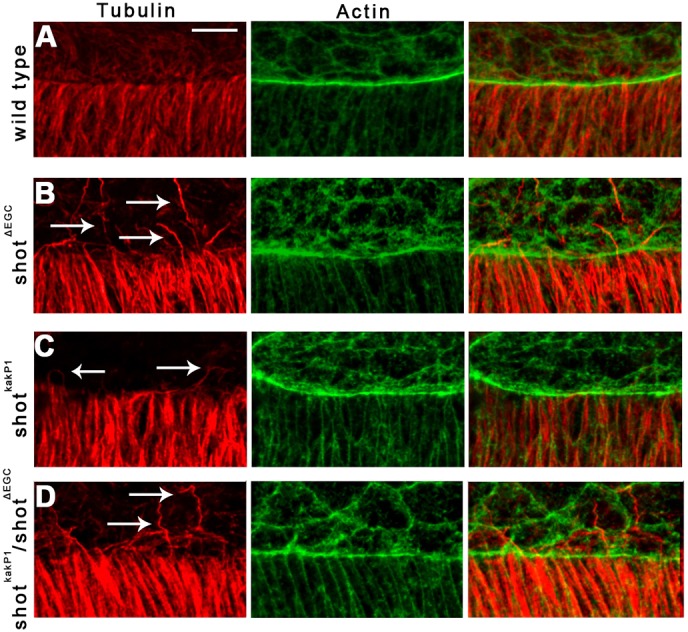
**Abnormal MTs at the leading edge of *shot^ΔEGC^* and *shot^kakP1^* mutant DME cells.** (A–D) Immunofluorescence staining of wild-type (A), *shot^ΔEGC^* (B), *shot^kakP1^* (C) and *shot^ΔEGC^*/*shot^kakP1^* mutant (D) embryos at the dorsal closure stage. Tubulin staining labels the MT network (red); actin is labeled with phalloidin (green). White arrows indicate abnormally long and curled MTs at the leading edge of mutant DME cells. Scale bar: 10 μm.

In order to better understand the role of Shot in MT regulation, we investigated the MT network of epithelial cells in isoform-specific *shot* mutants. In *shot^ΔEGC^* mutants, we detected abnormally long and bent MTs at the leading edge, indicating that the MT-binding activity of Shot is required for correct formation of MTs in DME cells (Fig. 5B). To further investigate the MT regulatory function of Shot in DME cells, we expressed various truncated versions of Shot in *shot^sf20^* null mutant embryos using the *en*-Gal4 driver, which drives the expression of the transgenes in only four-cell-wide stripes of the dorsally migrating epithelial sheets. This experimental design enabled us to compare *shot*-deficient cells with rescued cells in the same embryo (Fig. S3).

**Fig. 6.**
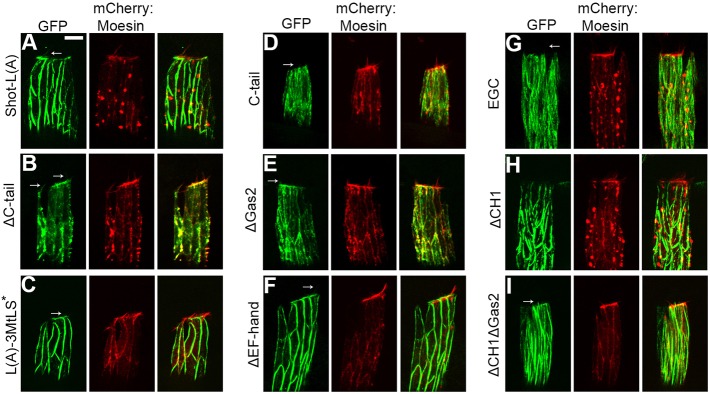
**Subcellular localization of Shot-L(A)–GFP and various truncated Shot protein forms in DME cells.** (A–I) Images show four-cell-wide *en*-Gal4-stripes of the epithelium in living embryos co-expressing GFP-tagged Shot protein variants and mCherry–Moesin. Localization of the Shot protein variants to the protrusions is indicated by arrows. Scale bar: 5 μm.

**Fig. 7.**
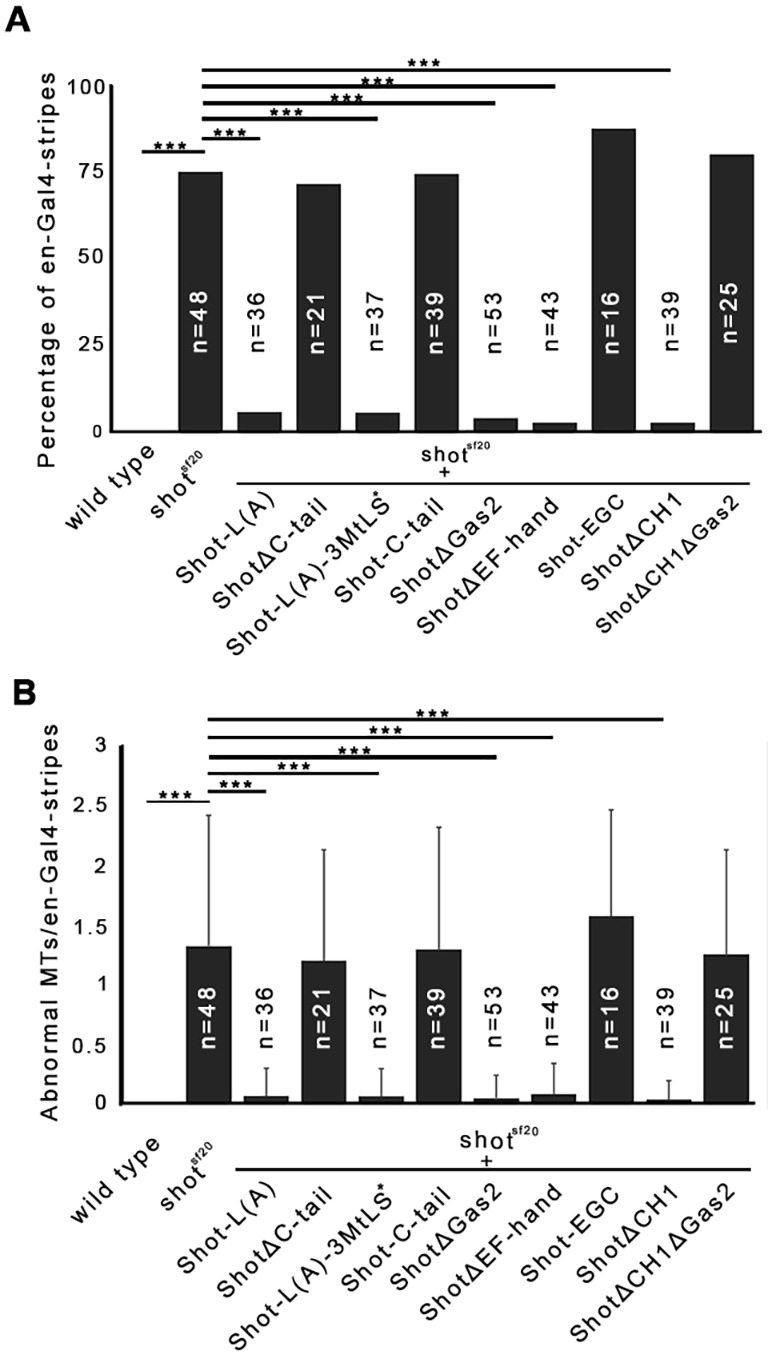
**Actin- and MT-binding activities of Shot are simultaneously required for proper MT organization in DME cells.** (A,B) Diagrams showing rescue of abnormal MTs protruding at the leading edge of DME cells of *shot^sf20^* mutant embryos expressing various GFP-tagged *shot* rescue constructs in four-cell-wide *en*-Gal4-stripes. (A) Percentage of *en*-Gal4-stripes with abnormally protruding MTs. ***P<0.001 (χ^2^-test). (B) Average numbers of abnormal MTs at the leading edge of DME cells in *en*-Gal4-stripes. ***P<0.001 (*t*-test). The mean±s.d. is shown.

The long transgenic Shot protein version, Shot-L(A)–GFP, predominantly colocalized with actin at the cell cortex and in the protrusions, and accumulated at the dorsal actin cable. Shot-L(A)–GFP was faintly detectable along filamentous structures which may correspond to a subset of MTs (Fig. 6A; Movie 5). Shot-L(A)–GFP failed to accumulate at growing MT plus-tips (Fig. S4; Movie 6). Expression of the Shot-L(A)–GFP protein completely rescued the abnormal MT phenotype of the *shot^sf20^* null mutants as indicated by the absence of long and bent MTs protruding from rescued DME cells (Fig. 7; Fig. S3B).

The Shot protein variant lacking the C-tail domain (ShotΔC-tail–GFP) localized diffusely in the DME cells and faintly decorated the cortical actin network and protrusions (Fig. 6B; Movie 7). ShotΔC-tail–GFP rescued abnormal MTs, indicating that plus-tip binding of Shot is not required for MT stabilization (Fig. 7; Fig. S3C). This result was confirmed by the expression of the Shot protein mutated exclusively for the EB1 interaction motifs [Shot-L(A)-3MtLS*–GFP], which localized similarly to Shot-L(A)–GFP and rescued the MT abnormalities of *shot* null mutants (Figs 6C, 7; Movie 8, Fig. S3D). Consistent with this finding, the C-terminal MT plus-tip-interacting domain of Shot (Shot-C-tail–GFP) on its own showed a localization in DME cells consistent with its previously reported ability to associate with the MT plus tips (Fig. 6D; Movie 9) (Alves-Silva et al., 2012; Applewhite et al., 2010). The expression of Shot-C-tail–GFP failed to rescue abnormal MTs (Fig. 7; Fig. S3E).

To reveal additional domains regulating MT organization, Shot protein lacking the Gas2 domain was expressed in epithelial cells. ShotΔGas2–GFP lost its ability to bind along the MT lattice and localized to the MT plus-tips and to the actin-rich cell cortex, indicating that the Gas2 domain inhibits the C-tail-mediated MT-tip binding of Shot (Fig. 6E; Movie 10). Expression of the ShotΔGas2 protein rescued the *shot^sf20^* null mutant MT phenotype, suggesting that the Gas2 domain is dispensable for MT stabilization (Fig. 7; Fig. S3F). The Shot protein version lacking the EF-hand domain (ShotΔEF-hand–GFP) localized similarly to the long Shot-L(A)–GFP and rescued abnormal MTs found in the null mutant cells (Figs 6F, 7; Movie 11, Fig. S3G). The transgenic *shot* construct composed of the EF-hand, Gas2 and C-tail domains alone (Shot-EGC–GFP) displayed a strong localization along the MT lattice but failed to rescue the abnormal MT phenotype (Fig. 6G, 7; Movie 12, Fig. S3H). Taken together, none of the three C-terminal domains seemed to be individually essential for MT stabilization, apparently contrasting with the results obtained with the *shot^ΔEGC^* mutant embryos. A possible explanation for this phenomenon could be that, at the leading edge, the Gas2 and C-tail domains of Shot mutually substitute for each other in MT regulation, suggesting that these domains have redundant functions in DME cells. In summary, we conclude that the MT-binding activity of Shot is required but is not sufficient for MT stabilization.

### Actin- and MT-binding domains of Shot organize the epithelial MT network

Therefore, to identify additional domains involved in MT stabilization, we tested the requirement for Shot's actin-binding activity by using *shot^kakP1^*, an isoform-specific mutant allele. In *shot^kakP1^* mutant DME cells, abnormally long and bent MTs were found, revealing that the actin-binding activity of Shot is required for MT stabilization (Fig. 5C). To further investigate the role of the actin-binding activity of Shot, ShotΔCH1–GFP, a Shot isoform lacking the CH1 domain and therefore actin-binding ability, was expressed in DME cells. The truncated protein lost its association with actin and localized intensely along the MTs in the cell body, indicating that the CH1 domain exerts an inhibitory effect on the ability of Shot to bind MTs (Fig. 6H; Movie 13). MTs decorated with ShotΔCH1–GFP were abnormally bundled and curved, raising the possibility that Gal4-driven overexpression of the truncated ShotΔCH1–GFP protein has a dominant gain-of-function effect on MT organization. No abnormally protruding MTs were observed at the leading edge of *shot^sf20^* null mutant in DME cells expressing ShotΔCH1–GFP (Fig. 7; Fig. S3I). The results obtained with the transgenic ShotΔCH1–GFP protein contrast with our previous observations on *shot^kakP1^* mutants, which display abnormal MTs protruding from the leading edge. The apparent capacity of the transgenic protein to rescue the phenotypes could arise from an artificial dominant effect caused by the overexpression of ShotΔCH1–GFP. Consistent with this idea, neuronal overexpression or a C-terminal truncation of Shot has been shown to dominantly cause alteration in MT network organization (Lee et al., 2016; Sánchez-Soriano et al., 2010). In DME cells, upon overexpression of ShotΔCH1–GFP, the truncated protein inhibits the formation of protruding MTs at the leading edge; however, it does not restore wild-type MT organization. Taken together, based on the *shot^kakP1^* mutant phenotype, we conclude that the actin-binding activity of Shot is required for MT stabilization.

The involvement of the actin- and MT-binding activities of Shot was further investigated by analyzing the ShotΔCH1ΔGas2–GFP variant lacking both the CH1 and the Gas2 domains. ShotΔCH1ΔGas2–GFP localized along the MT lattice but it was not capable of rescuing MT organization defects at the leading edge (Figs 6I, 7; Movie 14, Fig. S3J). Lack of rescue indicates that the actin-binding and the Gas2-mediated MT-binding activities of Shot are required for proper MT organization in the epithelial cells. Furthermore, these activities have to be simultaneously present in the same Shot molecule, as indicated by the abnormal MTs found at the leading edge of DME cells in *shot^ΔECG^*/*shot^kakP1^* embryos (Fig. 5D). In summary, Shot functions as an actin–MT crosslinker to ensure proper MT regulation at the leading edge of DME cells.

### Shot promotes filopodia formation at the leading edge of the epithelial cells

Our previous experiments revealed that both the actin and the MT regulatory activities of Shot function in DME cells are required to ensure proper MT organization and dorsal closure. Restoration of wild-type MT organization in *shot* mutants, however, was not sufficient for proper zippering, suggesting that an additional activity of Shot might be required for dorsal closure. Therefore, we investigated the actin network of DME cells by analyzing actin accumulation and protrusion formation at their leading edge.

**Fig. 8.**
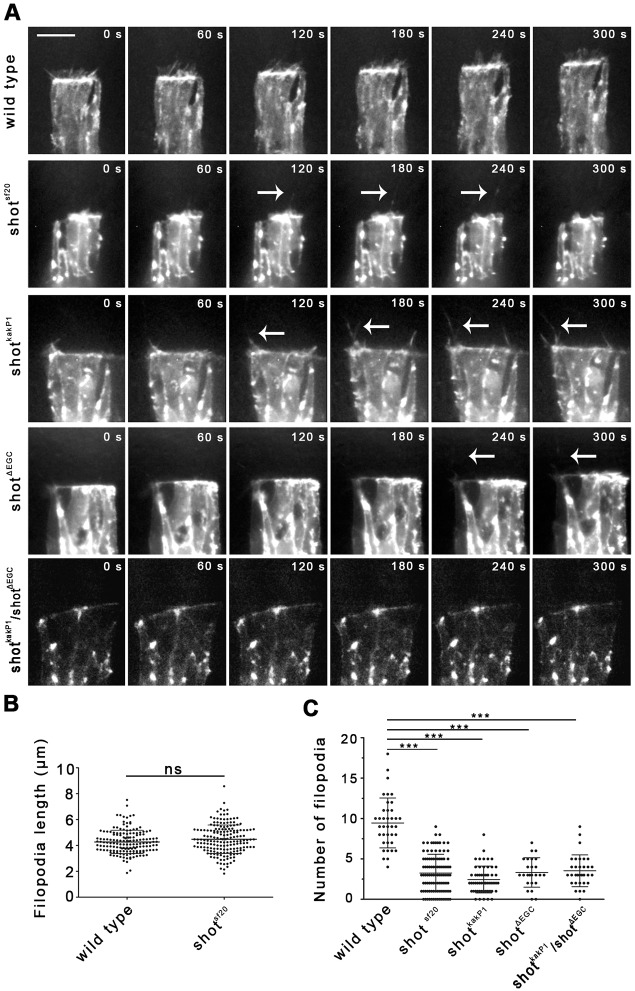
**Shot regulates filopodia formation.** (A) Movies showing DME cell protrusion dynamics in wild-type and *shot* mutant embryos expressing actin–EGFP. White arrows indicate long protrusions faintly decorated with actin–EGFP. Scale bar: 10 μm. (B,C) Quantification of filopodial dynamics in wild-type and *shot* mutants. Maximal length of filopodia (B) and the number of filopodia formed in 15 min (C) were measured in DME cells. In *shot* mutants, the number of filopodia decreased, whereas filopodial length remained unaffected. In B and C, the mean±s.d. is shown. ***P<0.001; ns, not significant (*t*-test).

In wild-type embryos, actin cables accumulate in epithelial cells at the leading edges. In fixed *shot^sf20^* null mutant and *shot^kakP1^* isoform specific mutant epithelia, normal actin accumulation was detected by phalloidin labeling (Figs 3B, 5C). In addition to accumulating actin, DME cells extend dynamic actin-based cell protrusions, such as lamellipodia and filopodia, at their leading edges. To identify the activity of Shot required for protrusion-mediated zippering, we analyzed the protrusion dynamics of DME cells by live imaging of *shot^sf20^* null mutants. Under the control of an *en*-Gal4 driver, mutant embryos expressed an actin–EGFP fusion protein, which specifically labeled the actin structures of the epithelial cells (Fig. 8A; Movie 15). In *shot^sf20^* embryos, in addition to normal looking filopodia and lamellipodia, we frequently observed long protrusions extending even 10 μm above the amnioserosa (Fig. 8A; Movie 15). These protrusions were faintly decorated with actin–EGFP and might correspond to the cell extensions containing abnormally long and bent MTs. Dynamic parameters such as length and the number of filopodia were measured. Although the filopodia length of *shot^sf20^* null mutant DME cells was normal, the number of filopodia was reduced, indicating that Shot is involved in regulating filopodia formation (Fig. 8B,C). This phenotype was a direct consequence of the loss of the actin- and MT-binding activities in Shot, as indicated by the reduced number of filopodia in *shot^kakP1^* and *shot^ΔEGC^* mutant cells (Fig. 8A,C). Furthermore, and supporting a requirement for crosslinking activity of Shot, the MT- and actin-binding domains have to be simultaneously present in the same Shot molecule as indicated by the reduced number of filopodia found at the leading edge of DME cells in *shot^ΔECG^*/*shot^kakP1^* transheterozygous embryos (Fig. 8A,C).

## DISCUSSION

Shot is a key structural component of the cytoskeleton and has been demonstrated to be involved in several regulatory processes leading to rapid changes in cellular morphology. Here, we show that the cooperative functioning of actin and MTs is essential for efficient dorsal closure, and their activities are coordinated by Shot. We describe two functions of Shot in organizing the MT network of DME cells and in regulating zippering by promoting filopodia formation.

### Shot regulates MT organization in DME cells

Based on their differential distribution and stability, we discriminate three subsets of MTs in DME cells. MTs of the first subset contain acetylated tubulin, a post-translational modification commonly associated with more stable MTs, which turn over slowly (Asthana et al., 2013; Matov et al., 2010; Matsuyama et al., 2002; Tran et al., 2007; Webster and Borisy, 1989; Zilberman et al., 2009). In DME cells, these MTs may correspond to the immobile fraction of tubulin found in FRAP experiments. The low immobile fraction indicates that only a small fraction of the MTs (∼one in five) belong to the stable MT subset. Acetylated MTs are concentrated at the apical surface of the cell body and are organized into parallel bundles. This MT arrangement is specific for the closure stage and seems to be independent of Shot activity, as indicated by the proper distribution of acetylated MTs in *shot* mutants. Furthermore, the immobile fraction of tubulin is not influenced by the *shot* null mutation. Thus, although Shot has been reported to influence MT stability in several cellular contexts, MT stabilization reflected by tubulin acetylation is insensitive to *shot* activity in DME cells.

MTs of second subset are the dynamic MTs found in the cell body. These MTs are aligned in an antiparallel manner in stable bundles and grow towards either the dorsal or the ventral periphery of DME cells. A possible role for Shot could be the guidance of MT growth along existing cytoskeletal filaments in a predefined pattern. Indeed, *in vitro*, parallel actin arrays can globally organize MT growth in a spectraplakin-dependent manner resulting in a parallel alignment of MTs (Preciado López et al., 2014). In neurons and *in vitro* assays, the MT guidance activity of Shot requires C-tail-mediated plus-end-binding and MtLS-motif–EB1 interaction (Alves-Silva et al., 2012; Preciado López et al., 2014). In DME cells, however, these interactions are dispensable for the organization of proper MT architecture, indicating that Shot does not play a prominent role in MT guidance in this cellular context. This conclusion is supported by the wild-type growth tracks observed by direct visualization of growing plus-tips in *shot* mutant DME cells. Thus, we hypothesize that additional, motor-driven guidance mechanisms are responsible for the establishment of the antiparallel arrangement of dynamic MTs (Akhmanova and Steinmetz, 2015; Chen et al., 2014; Doodhi et al., 2014; Mattie et al., 2010). Therefore, dynamic MTs in DME cells grow along each other or along acetylated stable MTs.

In the cell body of *shot* mutant DME cells, a slight increase in MT growth rate was detected indicating that Shot inhibits MT polymerization. This function of Shot is reflected by the faster turnover of tubulin measured by FRAP in *shot* mutant DME cells. A similar role for spectraplakins in MT growth speed regulation has been demonstrated in cultured *Drosophila* primary neurons and in human U2OS cells (Alves-Silva et al., 2012; Nishimura et al., 2012). Upon reaching the cell cortex, fast growing MTs of *shot* mutant DME cells continue to grow and either push out the cell cortex or bend along the lattice. The abnormal bending of the MTs could be responsible for the disorganized appearance of the MT network in the cell body of the *shot* mutant DME cells. Thus, Shot-mediated regulation of plus-end dynamics at the cell cortex contributes to the global organization of the MT network in the cell body.

MTs of the third subset reach the leading edge with their growing plus-ends and grow into protrusions. This fraction of MTs is severely affected by *shot* mutations: MTs are abnormally long and are frequently bent or curled. Similar MTs have been observed upon depletion of *shot* or ACF7 at the cell cortex in many other cell types, such as mammalian keratinocytes, endodermal cells, *Drosophila* S2 cells and neurons (Applewhite et al., 2010; Kodama et al., 2003; Sanchez-Soriano et al., 2009; Wu et al., 2008). Here, we show that, in addition to the Gas2-mediated MT association, Shot has to bind actin filaments simultaneously to regulate MT behavior. This observation is consistent with the hypothesis that bending of MTs occurs when they are not captured in the cortical actin network (Applewhite et al., 2010, 2013; Gierke and Wittmann, 2012). For cross-linking-mediated stabilization of MTs at the DME cell periphery, Shot associates with the MT lattice via its Gas2 domain and with cortical actin through its CH1 domain. Cortical tethering of MTs enables spatial control of MT dynamics by localized modification of plus-tip components, which promotes further cytoskeletal rearrangements required for the dorsal closure process. It remains to be determined what these cytoskeletal mechanisms are, but possible processes are protrusion formation or regulation of cell adhesion dynamics.

### Shot promotes zippering by regulating protrusion formation

Depending on domain composition and cellular context, spectraplakin isoforms are able to regulate actin and MT networks separately or coordinate their interactions by simultaneously binding to both filament systems. As distinct steps of dorsal closure involve both actin- and MT-based mechanisms, there are many potential points for Shot to regulate the closure process.

Dorsal-ward movement of the epithelial sheets is mainly driven by actomyosin contraction-generated forces in the amnioserosa (Ducuing and Vincent, 2016; Pasakarnis et al., 2016). Dorsal-ward displacement of the epithelial fronts is unaffected in *shot* mutants, indicating that *shot* is not required for the regulation of actin-based processes at this stage of the closure.

The last sealing step of the closure, zippering of the hole, however, requires *shot* function. Two major cellular mechanisms have been shown to facilitate closure at this stage: formation of protrusions that establish initial contacts between the opposing DME cells at the onset of zippering and the resolution of overlapping lamellar regions between them after they have met (Eltsov et al., 2015). Our data suggest that Shot regulates zippering by promoting protrusion formation rather than by supporting rearrangement of the lamellar interaction surfaces between DME cells. Resolution of lamellar overlaps has been suggested to be driven by microtubule-generated forces (Eltsov et al., 2015). However, our rescue experiments demonstrated that in addition to its function in MT regulation, the actin-binding activity of Shot is also required for zippering. While we cannot exclude the direct involvement of the MT regulatory function of Shot in overlap rearrangement, Shot-mediated stabilization of MTs alone is clearly not sufficient to ensure proper zippering.

In DME cells, proper protrusion dynamics requires both actin and MT activities (Eltsov et al., 2015; Hakeda-Suzuki et al., 2002; Harden et al., 1999; Jankovics and Brunner, 2006; Woolner et al., 2005). Here, we show that the coordinated action of the two filament types is required for proper filopodia function, and this coordination is orchestrated by Shot. In mediating zippering, the actin- and MT-binding activities of Shot are simultaneously required in the same molecule, indicating that Shot acts as a MT–actin crosslinker in this process. In DME cells, close alignment of MTs and actin filaments has been found exclusively in filopodia, thus, Shot exerts its crosslinking function in cell protrusions. This conclusion is supported by the reduction of the number of protrusions in *shot* mutants. We hypothesize that lack of actin-MT crosslinking causes abnormal protrusion formation in *shot* mutants, which in turn leads to inefficient zippering.

How Shot contributes to protrusion dynamics in DME cells remains elusive. In neuronal growth cones, the interaction of EF-hand domain of Shot with Kra (also known as eIF5C) has been described to be essential for filopodia formation (Sanchez-Soriano et al., 2009). In DME cells, however, the EF-hand domain is dispensable for dorsal closure, but the actin- and MT-binding functions of Shot are required for filopodia-mediated zippering. The differences in domain requirement indicate that Shot can promote protrusion dynamics by distinct mechanisms in various cell types. In cultured mammalian cells, interaction of the Shot homolog ACF7 with ELMO1 (also known as DOCK180) has been reported to promote protrusion formation by coordination of localized MT stabilization and Rac GTPase activity (Margaron et al., 2013). Possibly, a similar mechanism works in DME cells in which the interaction of Shot with the ELMO complex (ELMO is known as Mbc in *Drosophila*) stabilizes MTs by crosslinking them with the actin network and targets Rac activation to the leading edge. In support of this hypothesis, Rac activation has been shown to be required for protrusion formation and efficient zippering (Hakeda-Suzuki et al., 2002; Woolner et al., 2005).

The effect of the complete lack of Shot function during dorsal closure is very subtle, which can be explained by two, not mutually exclusive, hypotheses. First, that many additional crosslinkers or crosslinking mechanisms may act in parallel with Shot. A possible redundant cytoskeletal linker might be Pigs, which has been demonstrated to have CH1, Gas2 and C-tail domains by which it binds both actin and MTs (Girdler et al., 2016). Second, that the function of Shot is restricted to supporting protrusion formation in DME cells. Inefficient protrusion formation in *shot* mutants does not abolish zippering but reduces its efficiency (Eltsov et al., 2015; Jankovics and Brunner, 2006).

## MATERIALS AND METHODS

### *Drosophila* stocks

OregonR was used as wild type. The ZASP52^ZCL423^ protein trap line was used to visualize the leading edge of the DME cells (Morin et al., 2001; Stronach, 2014). *Pnr*-*GAL4* and *en*-GAL4 were used for inducible expression of the selected genes. For rescue experiments, the following transgenic constructs were used: Shot-L(A)–GFP, ΔEFhand–GFP [Shot-L(A)ΔEFhand–GFP], ΔGas2–GFP [Shot-L(A)ΔGas2–GFP], ΔCH1–GFP [Shot-L(C)–GFP]; these overexpression constructs were generated based on the *shot* mRNA annotated as transcript *shot-RE* in FlyBase (Lee and Kolodziej, 2002b). The encoded Shot protein contains all protein domains (CH1, CH2, plakin family domain, spectrin repeats, EF, Gas2 and the C-tail) apart from the plakin repeat region that is encoded by a single large exon and not contained within this mRNA (illustrated in Fig. 2C) (Röper and Brown, 2003; Röper et al., 2002). In addition, the following domain-deletion or single-domain transgenes derived from Shot-L(A)–GFP were used: ΔCH1ΔGas2–GFP [Shot-L(C)ΔGas2–GFP] (Lee and Kolodziej, 2002a), ΔC-tail–GFP [Shot-L(A)-ΔCtail–GFP], C-tail–GFP, Shot-L(A)-3MtLS*–GFP (Alves-Silva et al., 2012), EGC–GFP (Shot-EFhand-Gas2-Ctail–GFP) (Subramanian et al., 2003).

For loss-of-function analyses, we used *shot^sf20^*, *shot^kakP1^* and *shot^ΔEGC^* alleles and the Df(2R)BSC383 deficiency covering the *shot* locus.

Actin was visualized with UAS-actin–EGFP (Fulga and Rørth, 2002) or UAS-mCherry–Moesin (Millard and Martin, 2008), MT tips were labeled with UAS-EB1–EGFP (Jankovics and Brunner, 2006). For FRAP analysis, Ubi–β-Tubulin–EGFP was used (Inoue et al., 2004).

### DNA constructs and generation of the s*hot^ΔEGC^* allele

The novel *shot^ΔEGC^* allele was generated by the CRISPR/Cas9 genome editing method using vasa-Cas9 flies (Sebo et al., 2014). Target site-specific sequences for the *shot* gene were ligated into the pU6-BbsI-chiRNA plasmid (Table S2) (Gratz et al., 2013). A mixture of pU6-BbsI-target1 and pU6-BbsI-target2 plasmids was injected into *vasa*-Cas9 embryos. Novel *shot* mutations were selected by their lethal phenotype in a complementation analysis with the Df(2R)BSC383 deficiency. Deletion of the EF-hand and Gas2 domains was verified by sequencing.

### Live imaging, RNAi screening and time-lapse analysis of closure

Live imaging of embryos and RNAi-based loss-of-function genetic tests were performed as described in Jankovics et al. (2011). dsRNAs targeting 17 selected genes were generated by performing *in vitro* transcription on purified genomic DNA as a template (T7 RiboMAX Express, Promega) (Table S1).

For quantification of the zippering phenotype, the EGFP signal of the ZASP52^ZCL423^ protein trap line was imaged in *shot^sf20^*/*shot^sf20^* and *shot^kakP1^*/Df(2R)BSC383 and *shot^ΔEGC^*/Df(2R)BSC383 and *shot^kakP1^*/*shot^ΔEGC^* mutant embryos with a Leica TCS SP5 or a VisiScope spinning disc confocal microscope. For the rescue of closure defects, Shot variants were expressed in a *shot^sf20^* UAS-mCherry–Moesin/ Df(2R)BSC383; *pnr*-Gal4/+ mutant background. Measurement of the width and length of the dorsal opening was performed with ImageJ and analyzed with GraphPad Prism.

### Protrusion and MT dynamics measurements

For time-lapse imaging of protrusions, *shot^sf20^*/*shot^sf20^*, and Df(2R)BSC383/*shot^kakP1^*, and Df(2R)BSC383/*shot^ΔEGC^*, and *shot^kakP1^/shot^ΔEGC^* embryos were used. The number of filopodia extended in 15 min was calculated from measurements over more than 30 min. For measuring the MT growth rate and growth direction, *en*-Gal4/EB1–EGFP and *en*-Gal4, Df(2R)BSC383/*shot^sf20^*,UAS-EB1–EGFP embryos were imaged with an Olympus CellR microscope. Quantitative parameters were measured using ImageJ and analyzed with GraphPad Prism.

### FRAP

Fluorescence recovery experiments were performed on embryos expressing β-tubulin–EGFP (Inoue et al., 2004). FRAP experiments were performed with a Leica SP5 confocal microscope. A 405 nm laser was used to photobleach a 2 μm-wide stripe at the leading edge of the DME cells. Recovery after photobleaching was recorded for three minutes at 1 frame every 2 s. DME cells moving out of focus during image acquisition were ignored. Fluorescence recovery curves were analyzed using the easyFrap software as described in Bancaud et al. (2010). Statistical tests were performed with GraphPad Prism.

### Immunohistochemistry

Immunostainings were performed as described earlier (Jankovics and Brunner, 2006). Mouse-anti-Tubulin (1:10, E7, DSHB), rabbit-anti-EGFP (1:500, Life Technologies), mouse-anti-acetylated-tubulin (1:1000, 6-11B-1, Sigma), mouse-anti-FasciclinIII (1:20, 7G10, DSHB) and guinea pig anti-Shot-spectrin-repeats (1:1000) primary antibodies were used. To label actin, manually devitellinized embryos were incubated for one hour in Rhodamine–phalloidin (Molecular Probes). Specimens were examined with Leica TCS SP5 confocal microscope.
